# Differential Expression and Bioinformatics Analysis of CircRNA in PDGF-BB-Induced Vascular Smooth Muscle Cells

**DOI:** 10.3389/fgene.2020.00530

**Published:** 2020-05-29

**Authors:** Jiangtian Tian, Yahong Fu, Qi Li, Ying Xu, Xiangwen Xi, Yuqi Zheng, Li Yu, Zhuozhong Wang, Bo Yu, Jinwei Tian

**Affiliations:** ^1^Key Laboratory of Myocardial Ischemia, Chinese Ministry of Education, Harbin, China; ^2^Department of Cardiology, The Second Affiliated Hospital of Harbin Medical University, Harbin, China; ^3^Department of Pathology, Harbin Medical University, Harbin, China; ^4^Basic Medical College of Mudanjiang Medical College, Mudanjiang, China

**Keywords:** cardiovascular disease, RNA-seq, circRNA, VSMC, ceRNA

## Abstract

Atherosclerosis is mediated by various factors and plays an important pathological foundation for cardiovascular and cerebrovascular diseases. Abnormal vascular smooth muscle cells (VSMCs) proliferation and migration have an essential role in atherosclerotic lesion formation. Circular RNAs (circRNA) have been widely detected in different species and are closely related to various diseases. However, the expression profiles and molecular regulatory mechanisms of circRNAs in VSMCs are still unknown. We used high-throughput RNA-seq as well as bioinformatics tools to systematically analyze circRNA expression profiles in samples from different VSMC phenotypes. Polymerase chain reaction (PCR), Sanger sequencing, and qRT-PCR were performed for circRNA validation. A total of 22191 circRNAs corresponding to 6273 genes (host genes) in the platelet-derived growth factor (PDGF-BB) treated group, the blank control group or both groups, were detected, and 112 differentially expressed circRNAs were identified between the PDGF-BB treated and control groups, of which 59 were upregulated, and 53 were downregulated. We selected 9 circRNAs for evaluation of specific head-to-tail splicing, and 10 differentially expressed circRNAs between the two groups for qRT-PCR validation. Gene Ontology and Kyoto Encyclopedia of Genes and Genomes analyses enrichment analyses revealed that the parental genes of the circRNAs mainly participated in cardiac myofibril assembly and positive regulation of DNA-templated transcription, indicating that they might be involved in cardiovascular diseases. Finally, we constructed a circRNA-miRNA network based on the dysregulated circRNAs and VSMC-related microRNAs. Our study is the first to show the differential expression of circRNAs in PDGF-BB-induced VSMCs and may provide new ideas and targets for the prevention and therapy of vascular diseases.

## Introduction

Atherosclerosis (AS) is the main pathological basis of cardiovascular and cerebrovascular diseases and is mediated by various factors. With socioeconomic development, the morbidity and mortality of cardiovascular diseases are increasing worldwide; consequently, cardiovascular disease has become one of the important diseases threatening public health, and its causes and pathomechanism are not yet clear (Zhao et al., 2019). Vascular smooth muscle cells (VSMCs) are the major cellular components of the blood vessel wall, where they exist in a differentiated contractile phenotype to respond to arterial contraction and to produce extracellular matrix (ECM; Basatemur et al., 2019). Accumulating evidence shows that abnormal VSMC proliferation and migration have an essential role in atherosclerotic lesion formation. Genetic lineage tracing studies have illustrated that in atherosclerotic plaques, especially progressing plaques, extensive lipids are released by damaged or dying macrophages, and VSMCs. Then, accumulating lipid infiltration appeared in the center of the plaque, forming the necrotic core. VSMCs migrate and proliferate to the surrounding of the necrotic core and play an important role in creating a fibrous cap that stabilizes the atherosclerotic plaque (Feil et al., 2014; Misra et al., 2018). Moreover, VSMCs can differentiate into many other cell types found in the plaque core, suggesting that these cells might participate in multiple processes underlying atherosclerotic plaque stability (Tang et al., 2012; Ackers-Johnson et al., 2015; Durham et al., 2018; Wang et al., 2019).

An increasing body of evidence shows that the expression level of contractile SMC markers decreased is related with injury and inflammation, and which is associated with the reduced expression of MYOCD (a key factor regulating the contractile VSMC state in the development of plaques; Ackers-Johnson et al., 2015). *In vitro*, studies have demonstrated that SMCs, stimulated by growth factors, oxidative stress, and inflammatory cytokines, can phenotypically switch into proliferating, and/or migrating cells. Among them, platelet-derived growth factor (PDGF-BB) is considered to be one of the most effective mitogens in the proliferation and migration of VSMC, which can initiate various biological effects by activating intracellular signal transduction pathways and play a significant role in regulating the proliferation and migration of VSMC (Heldin and Westermark, 1999; Dzau et al., 2002; Shawky and Segar, 2017). Consequently, it will be necessary to find a new target to inhibit PDGF-mediated VSMC proliferation and migration which will exert an important therapeutic intervention in AS development.

Non-coding RNAs (ncRNAs) are a group of biomolecules acting as pivotal regulators that play powerful and diverse roles in pathological and physiological processes (de Almeida et al., 2016). Their gene expression patterns can also reveal changes in biological pathways that correlate with disease progression or even the risk of disease progression (Bayoumi et al., 2016; Zhang et al., 2019). Circular RNAs (circRNAs) are an emerging group of ncRNAs that are ubiquitous, stable, and evolutionarily conserved in eukaryotes (Memczak et al., 2013). Though the phenomenon of RNA cyclization was first reported in the 1970s (Sanger et al., 1976), circRNAs were considered as byproducts of aberrant splicing during transcription and remained underappreciated. As RNA sequencing technologies evolve, accompanied by the development of computational algorithms, numerous circRNAs have been discovered (Salzman et al., 2012). Notably, circRNAs have been found extensively in different species and are closely related to various diseases, including cardiopathy, which has a great impact on human health (Burd et al., 2010; Holdt et al., 2016; Huang et al., 2019). ncRNAs, especially microRNAs (miRNAs), and circRNAs, can function as competitive endogenous RNAs (ceRNAs) which can construt gene regulatory networks to regulate the expression of multiple genes with spatiotemporal specificity. Given the characteristics of ncRNAs, they could have great potential application in the treatment of diseases. However, the circRNA expression profiles and whether circRNAs participate in the regulatory of VSMCs still remain not clear. In the present study, we aimed to perform high-throughput RNA sequencing in paired PDGF-BB-treated VSMCs (PS) and a normal control group (NC) to investigate VSMC-specific circRNA profiles, as well as potential functional characterization of the representative candidate circRNAs. Our study is the first to show the differential expression of circRNAs in PDGF-BB-induced VSMCs and may provide new ideas and targets for the prevention and therapy of vascular diseases.

## Materials and Methods

### Cell Cultures

Primary human aortic smooth muscle cells (HASMCs; ScienCell, United States) were cultured with Smooth Muscle Cell Medium (SMCM; ScienCell, United States) in a humidified incubator with 5% CO_2_ at 37°C. HASMCs were inoculated at a density of 3 × 10^5^ cells/well in 6-well culture plates overnight. Before the subsequent experiments, cells were made quiescent by starvation for 24 h and then treated with 10 ng/ml platelet-derived growth factor (PDGF-BB; Sigma-Aldrich) for 48 h. The blank control group did not receive PDGF-BB. Each group had three samples.

### Immunofluorescent Staining

Human aortic smooth muscle cells treated with PDGF-BB and the contol were stained for a-SMA (abcam, United States). Brifly, cells of two groups were fixed with 4% paraformaldehyde for 20 min or overnight at 4°C, then, washed for 20 min in 0.5% Triton X-100 (Solarbio, China). Afterward, cells were blocked in 2% BSA for 30 min, then, washed in phosphate-buffered saline (PBS) for twice/10 min each time. Primary antibodies (a-SMA, 1:400 dilution) were added over night at 4°C, rinsed 3 times/10 min each time in PBS the following day, and subsequently the secondary antibody (1:1000 dilution) incubate at 37°C for 1 h. After incubation, the cells were washed 3 times with PBS, for 10 min each. Cell nucleus were labeled using DAPI for 5 min, then washed 3 times (5 min each) in PBS. Finally, cells were imaged with a fluorescent microscope.

### RNA Library Construction and Sequencing

According to manufacturer’s instructions, total RNA was isolated from each sample using TRIzol reagent (Invitrogen, Carlsbad, CA, United States). The amount and purity of the total RNA were quantified using NanoDrop ND-1000 (NanoDrop, Wilmington, DE, United States), and the integrity of total RNA was assessed by Agilent 2100 with RIN number >7.0. Then circRNA library was constructed. Firstly, approximately 5 μg RNA was subjected to ribosomal RNA depletion with the Ribo-Zero^TM^ rRNA Removal Kit (Illumina, San Diego, CA, United States). Secondly, linear RNAs were removed with RNase R (Epicentre Inc., Madison, WI, United States) to enrich circRNAs (3U RNase R for per μg RNA). Finally, the RNA fragmentation was obtained using divalent cations under high temperature for reverse-transcribed to generate first-stranded cDNA, then, the second-stranded DNAs were next synthesized with *Escherichia coli* DNA polymerase I, RNase H, and dUTP. To constrcut strand-specific cDNA, we added specificity terminal amino modification of the DNA fragment ends to prepare them for ligation to the adapters. After amplified by Polymerase chain reaction (PCR), the library was purified and the average insert size was 300 bp (± 50 bp). Finally, paired-end were sequenced on an Illumina HiSeq 4000 (LC Bio, China) according to the recommended protocol. The sequencing data used and/or analyzed during the current study are available in NCBI databases. (BioProject PRJNA607375).^1^

### Bioinformatics Analysis

Low-quality and undetermined bases was removed and sequence quality was verified using FastQC^2^. Then, we used Bowtie2 and Tophat2 to map reads to the reference genome. CIRCExplorer and TopHat-fusion were utilized for *de novo* assembly of the mapped reads to circRNA and recognizing back splicing reads in unmapped reads. All samples generated unique circRNA. The differentially expressed circRNAs with log2 (fold change) >1 or log2 (fold change) <−1 and with statistical significance (*p* value <0.05) by R package–edgeR were selected for further studies.

### CircRNA Validation by PCR

Polymerase chain reaction was used to validate the reliability of the high-throughput RNA sequencing data. A Transcriptor First Strand cDNA Synthesis Kit (Roche, Germany) was used for reverse transcription of circRNAs. According to manufacturer’s instructions, appropriate volume of master mix as well as RNA sample were prepared, then the reaction for reverse transcription was initiated at 25°C for 10 min, 55°C for 30 min, and 85°C for 5 min. Then, cDNA and gDNA templates were PCR amplified for 35 cycles using Taq PCR MasterMix (Tiangen, China) following the manufacturer’s protocol, and PCR products were visualized using 2% GelRed-stained agarose gel. To confirm the PCR results, we further performed Sanger sequencing to directly examine the PCR product. To verify the accuracy of the differential expression of circRNAs, qRT-PCR was conducted using a FastStart Universal SYBR Green Master Kit (Roche, Germany). Briefly, the first strand cDNA was synthesized using random hexamer primer and then amplified by SYBR Green Kit following the standard procedure that is denaturation 95°C (10 min) followed by amplification by a total of 40 cycles of 95°C (15 s) and 60°C (1 min) on an ABI7500 system (Applied Biosystems, Foster City, CA, United States). GAPDH was used as an internal control, and PCR primers are listed in Supplementary Table S1.

### GO and KEGG Pathway Analyses

The differentially expressed circRNA-host gene data were analyzed by the DAVID tool (V6.8; Huang da et al., 2009) with its GO function enrichment and KEGG pathway analyses. An enrichment gene count ≥2 and hypergeometric test significance threshold *P* value <0.05 were considered to indicate significant enrichment.

### Interaction Between CircRNA and miRNA

Vascular smooth muscle cell-associated miRNAs were selected from disease-miRNA interactions validated in previous studies (Leeper and Maegdefessel, 2018; Wang and Atanasov, 2019). For the obtained VSMC-related miRNAs, we predicted whether there was a regulatory relationship between them and the selected differentially expressed circRNAs. We used miRanda and TargetScan to predict the relationships between the VSMC-related miRNAs and the differentially expressed circRNAs, and the Cytoscape tool was used to construct a network map of target miRNAs and circRNAs.

### Statistical Analysis

Data were analyzed and visualized with SPSS 22.0 (IBM Corporation, Somers, NY, United States) and GraphPad Prism 5.0 (GraphPad Software, La Jolla, CA, United States). Data are presented as the mean ± standard deviation. Wilcoxon rank-sum test, Student’s *t*-test and fold change were used to analyze the significant differences between the sequencing data of samples. A *t* test was applied to compare qRT-PCR results. Differences with *p* <0.05 were considered statistically significant.

## Results

### CircRNA Expression Profiles in PDGF-BB-Treated VSMCs

Cells were treated with 10 ng/ml PDGF-BB (PS), and the blank control group did not receive PDGF-BB (NC). After stimulation for 48 h, morphological changes and the expression levels of SM22α and α-SMA in the two groups were detected and are shown in Supplementary Figures S1A–C. As expected, VSMCs tended to phenotypically switch to function as proliferative and/or migratory cells in response to stimulation by PDGF-BB (Heldin and Westermark, 1999; Allahverdian et al., 2018). Compared to the NC, the cell morphology became spindle-shaped and elongated, and the expression of differentiation-associated genes and proteins was decreased in the PS, suggesting that the cells had a stronger capacity for proliferation. In the meantime, 7 known SMC markers were detected in NC and the PS group and we added a heatmap of the differential mRNA expression between the two groups (Supplementary Figure S1D). The result showed that the synthesis markers of KRT8 and TLR4 were significantly increased and contractile markers including MYH11, SMTN, CNN1, SMM22α, and α-SMA were reduced in PDGF-BB treated HASMC, which corresponded to the privious study. These studies fully confirmed that the cells gain a proliferative phenotype after treatment with PDGF-BB.

**FIGURE 1:**
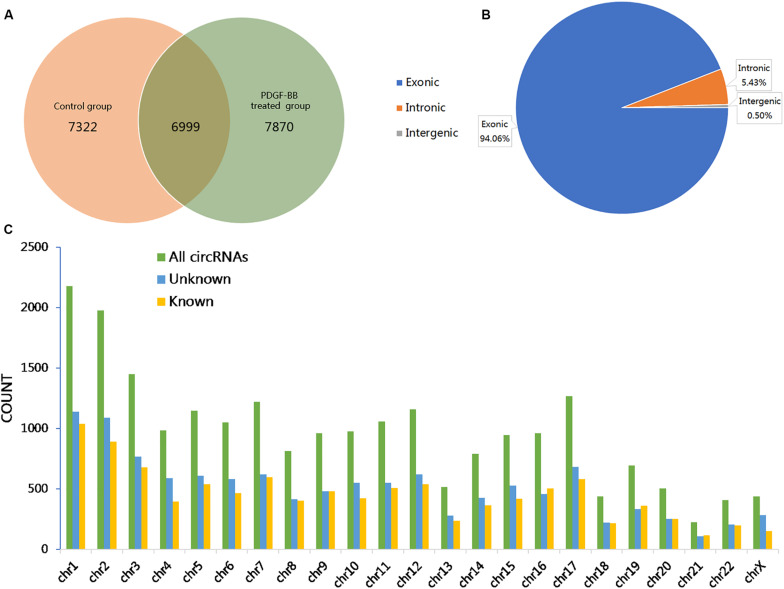
Overview of the identified circular RNAs (circRNAs) in PDGF-BB-treated VSMCs (PS) and controls (NC). **(A)** The Venn diagram shows the number of unique and common circRNAs in PS and NC. **(B)** The pie chart displays the ratio of circRNAs originating from exonic, intronic, and intergenic regions. **(C)** Chromosomal distribution of all identified circRNAs.

To investigate the effects of PDGF-BB on circRNA expression in VSMCs, two groups of HASMCs were prepared for high-throughput sequencing using an Illumina HiSeq 4000 (LC Bio, China). We used CIRCExplorer to *de novo* assembly of the mapped reads and to identify back splicing reads. The following criteria were restricted for circRNA identification: (1) mismatch ≤2; (2) back-spliced junction reads ≥1; and (3) two splice sites <100 kb apart on the genome. Accordingly, a total of 22191 circRNAs, corresponding to 6273 genes (host genes) in the PS, NC, or both groups, were detected, of which 7322 and 7870 circRNAs were specifically expressed in the NC and PS groups, respectively. A total of 6999 circRNAs were identified in both the PS and NC groups (Figure 1A). Further analysis revealed that three categories of circRNAs were represented: exons (94.06%), introns (5.43%), and intergenic regions (0.50%; Figure 1B). The results showed that the majority of the circRNAs originated from protein-coding exons. By analyzing the sequencing data, we identified 5794 circRNAs recorded in circBase^3^ and 16397 novel circRNAs that were discovered in this study. The chromosomal distribution of all circRNAs showed that these circRNAs were distributed on almost all human chromosomes (Figure 1C).

**FIGURE 2:**
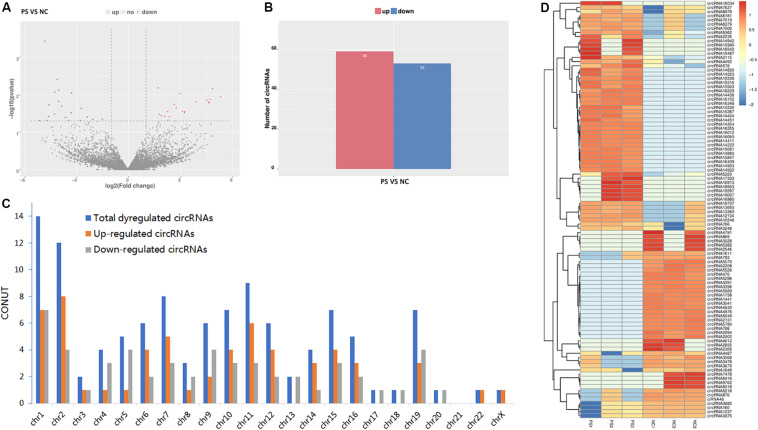
Differentially expressed (DE) circRNAs in PS and NC. **(A)** and **(B)** display the volcano plot and bar graph for the DE circRNAs in the samples of the two groups. **(C)** The chromosome distributions of differentially expressed circRNAs. **(D)** The DE circRNAs and samples are coclustered by hierarchical clustering analysis. The upper maps are based on DE circRNAs with log2 (fold change) >1 or log2 (fold change) <-1 and *p* value <0.05 for the comparisons of PS vs NC.

By the criteria of log2 (fold change) >1 or log2 (fold change) <−1 and *p* value <0.05, we identified 112 differentially expressed circRNAs between the PS and NC groups, of which 59 were upregulated, and 53 were downregulated. The DE circRNAs in the samples of the two groups are displayed with a Volcano plot, bar graph, and heatmap. Additionally, the chromosome distributions of the DE circRNAs are shown in the bar graph (Figures 2A–D). The top ten upregulated and downregulated circRNAs are listed in Table 1.

**TABLE 1:** Top ten upregulated and downregulated circRNAs.

CircRNA Name	Gene symbol	Regulation	Annotation	Chromosome	Fold change (log2)	*P* value
CircRNA12134	AC004922.1	Up	Exon	Chr7	5.312	0.011
CircRNA6181	SSH1	Up	Exon	Chr12	4.847	0.007
CircRNA7019	AP1G1	Up	Exon	Chr16	4.691	0.013
CircRNA7000	SNTB2	Up	Exon	Chr16	4.646	0.016
CircRNA8379	HMGCR	Up	Exon	Chr5	4.605	0.016
CiRNA10248	CDR1	Up	Exon	ChrX	4.509	0.014
CircRNA10737	HINFP	Up	Exon	Chr11	3.905	0.016
CircRNA13693	SOX13	Up	Exon	Chr1	3.238	0.028
CircRNA13360	GIGYF2	Up	Exon	Chr2	3.222	0.030
CircRNA8979	HPCAL1	Up	Exon	Chr2	3.111	0.028
CircRNA5297	TBCE	Down	Exon	Chr1	–5.159	0.049
CircRNA876	CCNY	Down	Exon	Chr10	–4.855	0.000
CircRNA198	PCCA	Down	Exon	Chr13	–4.714	0.049
CircRNA5218	NEK7	Down	Exon	Chr1	–4.629	0.039
CircRNA3568	PRELID2	Down	Exon	Chr5	–4.602	0.019
CircRNA3679	AFAP1	Down	Exon	Chr4	–4.283	0.033
CircRNA1611	IST1	Down	Exon	Chr16	–4.270	0.009
CiRNA46	SRF	Down	Intron	Chr6	–4.130	0.004
CircRNA4487	MITD1	Down	Exon	Chr2	–4.052	0.008
CircRNA793	ZMYND11	Down	Exon	Chr10	–4.046	0.015

### Validation of VSMC-Enriched CircRNAs

**FIGURE 3:**
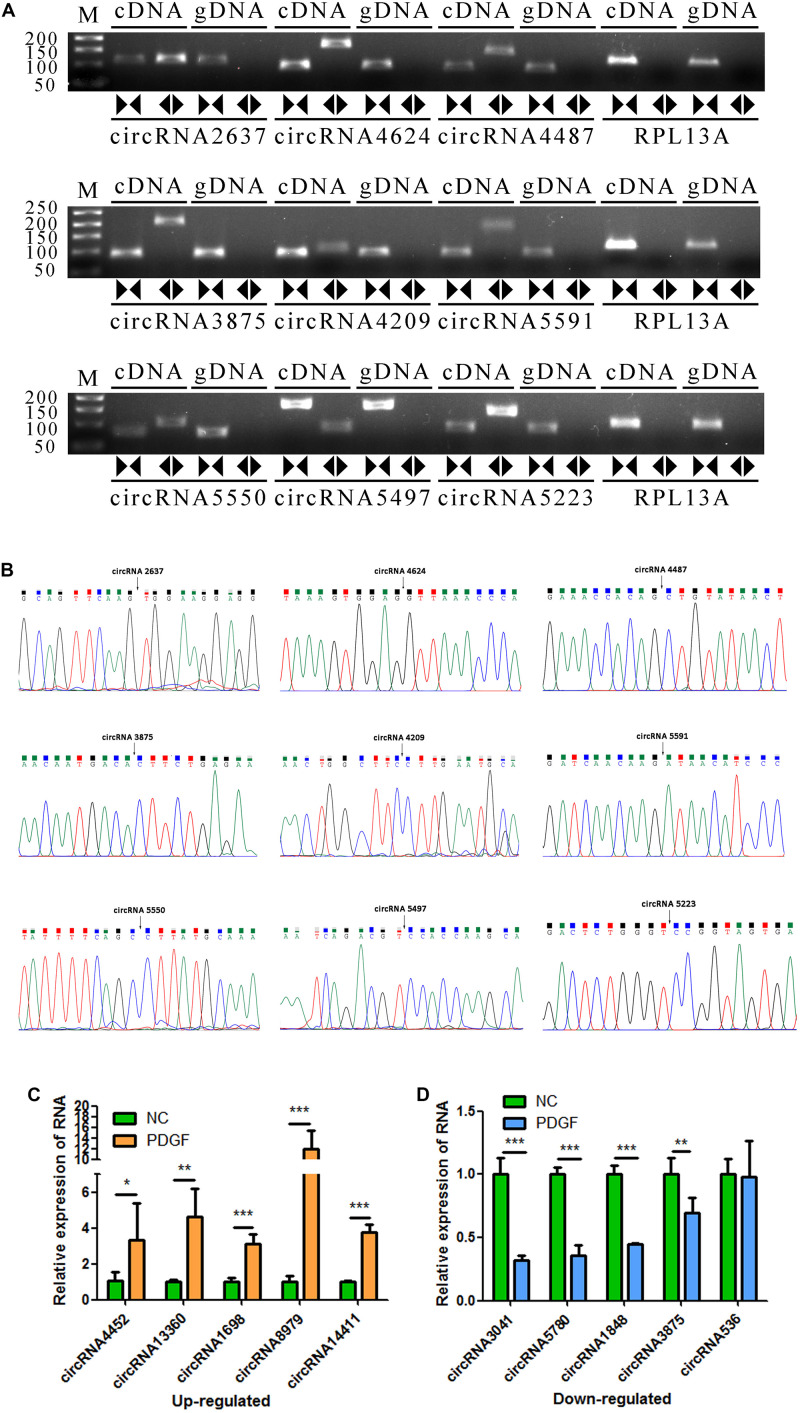
Validation of VSMC-enriched circRNAs. **(A)** Nine circRNAs were selected for validation experiments. RT-PCR with divergent (circular) and convergent (linear) primers was used to confirm the candidate circRNAs. Divergent (circular) primers (◀▶) successfully amplified a single fragment at the expected sizes from cDNA but not from genomic DNA (gDNA). Convergent (linear) primers (▶◀) could amplify from both cDNA and gDNA. **(B)** Sanger sequencing of the selected candidate circRNAs shows the back-splice junctions. **(C,D)** The relative expression levels of 10 DE circRNAs were determine by qRT-PCR. The data are presented as the mean ± SD, *n* = 3. *P <0.05, **P <0.01, and ***P <0.001.

To verify the accuracy of the RNA-seq data, 9 circRNAs (circRNA2637, circRNA4624, circRNA4487, circRNA3875, circRNA4209, circRNA5591, circRNA5550, circRNA5497, and circRNA5223) were randomly selected for validation experiments. We used PCR to evaluate specific head-to-tail splicing. First, divergent (circular) and convergent (linear) circRNA-specific primers were designed for RT-PCR to verify that the selected candidate RNAs are indeed circRNAs. The results showed that the divergent primers produced amplicons from RNA-derived samples and not from genomic DNA. The PCR products were visualized using 2% GelRed-stained agarose gel (Figure 3A). Then, Sanger sequencing was performed to validate the PCR product, and the head-to-tail splice junctions were identified, unambiguously demonstrating that the selected candidates were circRNAs (Figure 3B). Finally, ten circRNAs (threshold: a fold change greater than 1 and a *p* value less than 0.05 in two comparisons that were differentially expressed in the two groups) were selected for qRT-PCR validation, and three biological replicates were performed. The results showed that the expression levels of circRNA-4452, circRNA-13360, circRNA-1698, circRNA-8979, and circRNA-14411 were significantly upregulated, and the expression levels of circRNA-3041, circRNA-5780, circRNA-1848, and circRNA-3875 were significantly downregulated. Accordingly, Three of the candidate circRNAs were identified the sequence of head-to-tail splice junctions directly by Sanger sequencing of PCR amplicons (Supplementary Figure S2). The qRT-PCR assay results were consistent with our RNA-seq assay results, confirming the accuracy of sequencing. However, circRNA-536 was not consistently and/or significantly differentially expressed between the two groups (Figures 3C,D).

### GO and KEGG Analyses of the Dysregulated CircRNA Parental Genes

**FIGURE 4:**
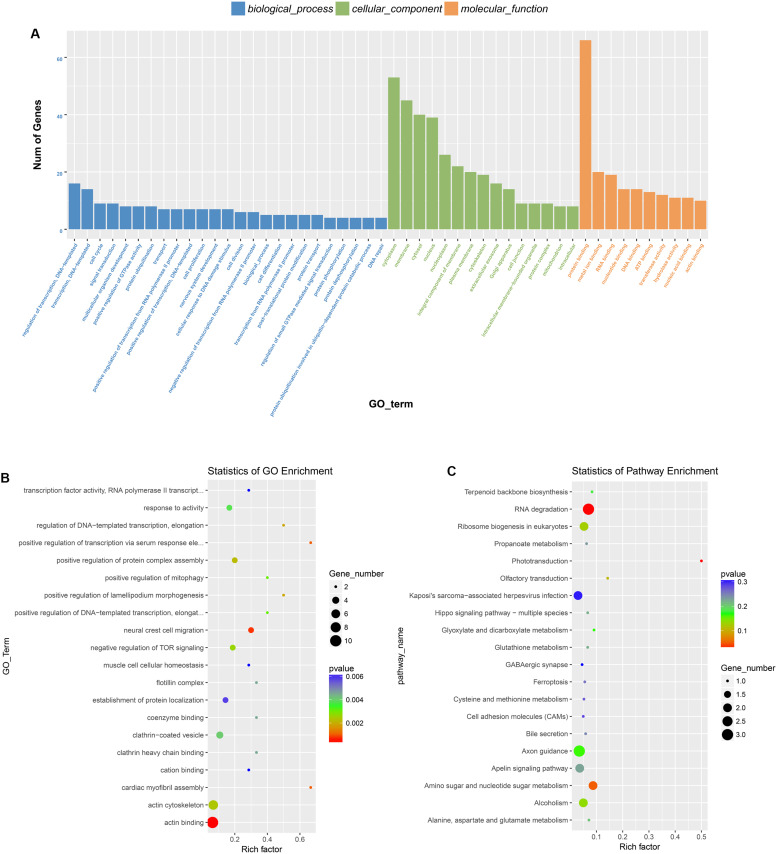
GO and KEGG pathway analysis of the parental genes of the identified circRNAs. **(A)** The top 25 GO BP, top 15 GO CC, and top 10 GO MF are displayed. **(B)** The top 20 most represented significant GO terms are displayed in a scatter plot. **(C)** The top 20 enriched KEGG pathways of the circRNA parental genes.

**FIGURE 5:**
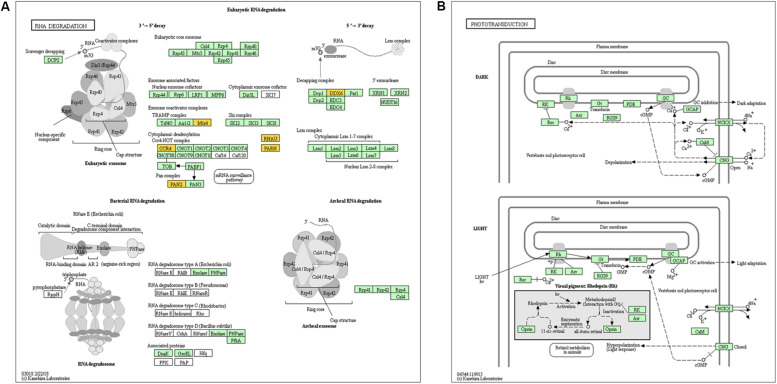
Genes mapped to KEGG pathways by pathway analysis. **(A)** and **(B)** show the signaling pathways “RNA degradation” and “phototransduction,” which were the most significant pathways.

Previous studies have demonstrated that circRNAs are closely related to their parental genes and have the ability to regulate their parental genes (Zhang et al., 2016; Wei et al., 2017). Thus, to further investigate the panorama of circRNA functions and interactions in VSMCs, we performed GO function enrichment analysis and KEGG pathway enrichment analysis based on the significantly differentially expressed circRNA host genes. The results of GO enrichment analysis showed that 782 GO BP (biological process), 198 GO CC (cellular component), and 241 GO MF (molecular function) terms were enriched. The top 25 (GO BP), top 15 (GO CC), and top 10 (GO MF) are displayed in Figure 4A. GO enrichment showed that 66 genes were enriched in protein binding, 53 genes were expressed in the cytoplasm, and 16 genes were mainly associated with regulation of transcription, DNA-templated. In the GO category “cellular component,” the most significant terms were actin cytoskeleton, clathrin-coated vesicle and flotillin complex, while in the GO category “molecular function,” the main molecular functions were actin binding, clathrin heavy chain binding, coenzyme binding, transcription factor activity, and RNA polymerase II transcription factor binding. In the GO category “biological process,” neural crest cell migration, positive regulation of transcription via serum response element binding, cardiac myofibril assembly, and positive regulation of DNA-templated transcription were the most representative significant terms. The top 20 GO terms are displayed in a scatter plot (Figure 4B). KEGG pathway enrichment analysis suggested that there were 81 signaling pathways related to the differentially expressed genes, among which the “RNA degradation,” and the “phototransduction” signaling pathways were the most significant pathways. The top 20 pathways are shown in Figure 4C. The two most significant pathways are displayed in Figure 5.

### Construction of a CircRNA and Disease-Related miRNA Network

**FIGURE 6:**
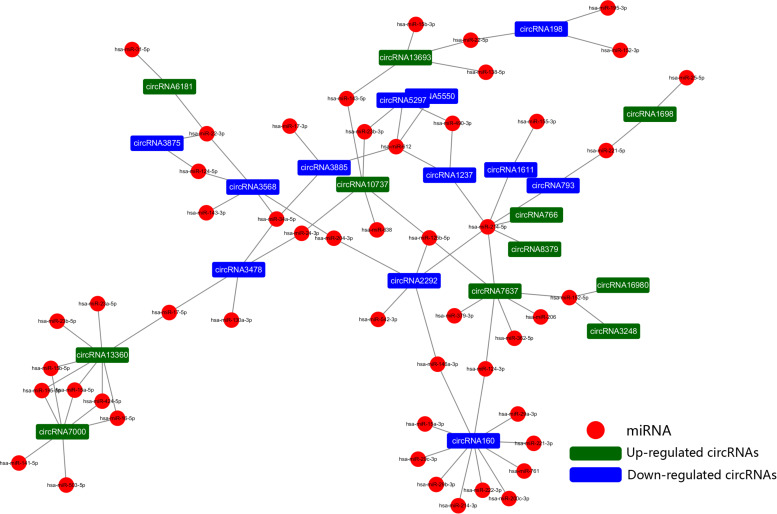
The circRNA-miRNA network. The generated network consists of 73 nodes (23 dysregulated circRNAs and 50 miRNAs) and 83 edges. According to the network, we predicted that 12 down- and 11 upregulated circRNAs have miRNA binding sites and could act as ceRNAs to regulate posttranscriptional gene expression.

To further explore the regulatory mechanism of the DE circRNAs and investigate the relationships between DE circRNAs and the function of VSMCs, we compared VSMC-associated miRNAs in validated disease-associated miRNA. A detailed list of the miRNAs is provided in Table 2. Based on the ceRNA regulatory mechanism, TargetScan, and miRanda software were used to analyze the miRNAs binding sites for DE circRNA. Finally, we constructed a circRNA-miRNA interaction network, 73 nodes (23 DE circRNAs and 50 miRNAs) and 83 edges were identified in the circRNA–miRNA network (Figure 6). According to the network, we predicted that 12 down- and 11 upregulated circRNAs have miRNA binding sites that could act as ceRNAs participating in the regulation of posttranscriptional gene expression. Notably, we found that circRNA160 (hsa_circ_0008776) showed the highest degree of connectivity in the network, with up to 11 miRNA binding sites, followed by circRNA13360 (hsa_circ_0003341), and circRNA7637 (hsa_circ_0001222), indicating their potential important function in VSMCs and cardiovascular disease.

**TABLE 2:** Detailed list of the VSMC-associated miRNAs.

miRNA	Target(s)	Role and function in SMC dynamics	References
miRNA-214	NCK associated protein 1 (NCKAP1)	Migration, Proliferation, and Neointima Hyperplasia	Afzal et al., 2016
miRNA-130a	MEOX1	Proliferation, migration	Wu et al., 2011
miRNA-675	PTEN	Proliferation	Lv et al., 2018
miR-221/-222	CDKN1B, CDKN1C	Proliferation, migration, and anti-apoptotic effects	Davis et al., 2009; Liu et al., 2009
miRNA-22-3p	High mobility group box-1 (HMGB1)	Proliferation and Migration and Neointimal Hyperplasia	Huang et al., 2017
miRNA-23b	The transcription factor forkhead box O4 (FoxO4)	Phenotypic switching	Iaconetti et al., 2015
miRNA-195	The Cdc42, cyclin D1, and fibroblast growth factor 1 (FGF1) genes	Regulate cell phenotype and prevents neointimal formation.	Wang et al., 2012
miR-206	ARF6, SLC8A1	Differentiation	Lin et al., 2016
miRNA-34a	Neurogenic locus notch homolog protein-1 (Notch1)	Proliferation and migration	Chen et al., 2015
miR-146a	KLF4/5	Differentiation, proliferation	Sun et al., 2011

## Discussion

AS is a complex pathological process characterized by endothelial dysfunction, lipid infiltration, oxidative stress, inflammation, cell proliferation, and apoptosis (Weber and Noels, 2011; Lu and Daugherty, 2015). During the development of AS, the arterial wall is stimulated by multiple harmful conditions, such as hyperlipidemia, hypertension, diabetes, smoking, homocysteinemia, and other agents that may respond to multiple signaling molecules that interact with the lining of the endothelium, altering the homeostatic condition of the arterial wall and resulting in the migration and proliferation of VSMCs within the lesions (Ross, 1995). It has been considered that phenotypic transformation of VSMCs is an important contributor to vascular disease development, including the pathologic process of atherosclerotic plaque development (Bennett et al., 2016). In recent years, through the combination of high-throughput sequencing and bioinformatics analysis, an increasing number of RNA categories and important potential targets for gene therapy have been discovered (Dixon et al., 2005; Guo et al., 2014). A variety of studies confirmed a strongly association between circRNAs and cardiovascular disease (Tan et al., 2017). Holdt et al. (2016) demonstrated that circANRIL could induce cell apoptosis and inhibit cell proliferation through inducing nucleolar stress and p53 activation. Huang et al. (2019) found that circRNA Nfix regulated by superenhancers (SEs) acts as a pivotal element in regulating cardiac regeneration. However, the expression profiles of circRNAs in different VSMC phenotypes are not yet known.

In this study, it is the first time to show the differential expression of circRNAs in two groups of VSMCs. We screened circRNA profiles to identify the dynamically changed circRNAs in order to discover pivotal biomarkers for vascular biology. A total of 22191 circRNAs were identified in both the PS and NC groups. Currently, five classes of circRNAs have been detected, circular RNA genomes (viroid and hepatitis delta virus circles), circular RNA from introns, circular RNA intermediates in RNA processing reactions, circRNA from exons, and circRNA in archaea with snRNP functions (Lasda and Parker, 2014). Further analysis of the present study revealed that three categories of circRNAs were represented, among which exon-based circRNAs were the overwhelming majority (94.06%). Using a calculation of FDR to analyze the difference molecules of 22191 circRNAs, we found that the positive results were few. Based on the identification of two groups of cell phenotypes, we think that the screening algorithm we used may be too strict, and the results may lose a large number of true positive results. Therefore, we changed the screening criteria, using the criteria of log2 (fold change) >1 or log2 (fold change) <−1 and *P* value <0.05 as previous studies (Dang et al., 2017; Xu et al., 2018; Hu et al., 2019) to screen out 112 different expression circRNAs between PS and NC, and then randomly selected 10 circRNAs for verification experiment. On the basis of verification, we calculated the false positive rate (FPR) to generate the *Q* value. The false-positive rate is only 10%, which shows that the range of difference we selected is of great reference value. And the PCR results were quite consistent with the sequencing results, confirming the high reliability of the high-throughput sequencing data. Cluster analysis was used to display the differences of circRNA expression between the PDGF-treated and control groups. From the analysis, we found that the expression of circRNAs showed a significant difference between the treatment group compared to the control group. The results showed that different circRNAs are turned off and on in different cell states at different times and are involved in numerous metabolic processes. Such circRNAs may become novel prognostic markers for diseases.

GO and KEGG pathway enrichment analyses of the host genes we detected showed that the parental genes of the circRNAs mainly participate in cardiac myofibril assembly and positive regulation of DNA-templated transcription, indicating that they might be involved in cardiovascular diseases. Moreover, according to the BP, CC, and MF terms with substantial enrichment, the genes were mainly associated with gene expression at the transcriptional or posttranscription level in the cytoplasm. The “RNA degradation” pathway is an important signaling pathway associated with many biological processes in eukaryotes.

A variety of studies have revealed that circRNAs can function as sponges for related miRNAs, thus playing a vital regulatory role in influencing physiological processes as well as various diseases, including cardiovascular disease, of which miRNAs act as indispensable bridges joining various RNAs (Salmena et al., 2011; Tay et al., 2014). The circRNA-miRNA network has been proven to be a widely accepted mechanism of gene expression regulation. Hence, a circRNA-miRNA network was constructed based on the dysregulated circRNAs and VSMC-related miRNAs. Based on ceRNA theory, circRNAs containing MRE binding site could act as a miRNA sponge to regulate gene expression, in other words, there was a negative correlation in the expression of circRNA and miRNA. Among the altered circRNAs in the network, we found that circRNA160 (hsa_circ_0008776) which is significantly downregulated after PDGF-BB treated VSMC, showed the highest degree of connectivity throughout the network, with up to 11 miRNA binding sites. Among them, it is demonstrated that the expression of miR-221-3p, miR-222-3p, and miR-146a-3p were increased by growth stimulators and overexpression these miRNAs could dramatically enhance VSMC proliferation, growth, and migration (Liu et al., 2009; Sun et al., 2011; Li et al., 2019). In addition, circRNA13360 (hsa_circ_0003341) was upregulated in PS group and has been predicted to have an MRE binding site and may act as a miRNA sponge for miR-23b-5p and miR-424-5p et al., studies suggested that overexpression of miR-23b and miR-424 inhibited VSMC proliferation and migration (Merlet et al., 2013; Iaconetti et al., 2015). Since the function of these RNAs in the network has been partially demonstrated and our results are in line with previous studies, suggesting a potential vital function of circRNAs in the pathology of AS. Additional analyses revealed that circRNA160, encoded by the parental gene THSD1 thrombospondin type I domain (1), was significantly downregulated in PDGF-BB-treated VSMCs. Although the regulatory roles of circRNA160 in the proliferation or migration of VSMCs remain largely unknown, THSD1 is a novel regulator during vascular development and functions to protect the intraplaque microvasculature and prevent hemorrhaging in advanced atherosclerotic lesions (Haasdijk et al., 2016). The expression of THSD1 could be regulated, depending on activation by multiple microenvironmental factors. However, studies that elucidate the regulation of THSD1 are still lacking, and whether circRNA160 can regulate the transcription of its parental genes remains to be further studied.

In this study, we aimed to discover pivotal biomarkers for vascular biology to provide evidence supporting molecular therapy for the diseases. But objectively, circRNAs have diverse functions, apart from miRNA sponging, they can directly influence or control mRNA transcripts or effect protein translation and function. In addition, some of them have the ability to translate peptides (Lasda and Parker, 2014; Du et al., 2017; Pamudurti et al., 2017). However, the data information in our study could provide clues for further research. We could conduct in-depth studies to explore the regulatory role in disease development, providing important targets for disease treatment or diagnosis.

## Conclusion

In summary, the proliferation and migration of VSMCs are important contributing factors to vascular disease development, including the pathologic process of atherosclerotic plaque progression. In this study, we identified the differential expression of circRNA in PDGF-BB-induced VSMCs. A circRNA-miRNA network was constructed, and bioinformatics analysis suggested that circRNAs may play vital roles in the pathology of AS, especially at the posttranscriptional level. This is only the beginning toward a better understanding of the roles of circRNAs in VSMCs, and more functional experiments are still needed to confirm the precise molecular regulatory mechanisms of circRNA functions.

## Data Availability Statement

The sequencing data used during the current study are available in NCBI databases (BioProject PRJNA607375).

## Author Contributions

JiaT and YF performed the bioinformatics analyses and wrote the manuscript. QL and YX designed the primers and conducted qRT-PCR assays. XX and YZ performed the cell experiments. LY and ZW analyzed the data. JinT and BY conceived, designed, and supervised the study. All authors read and approved the final manuscript.

## Conflict of Interest

The authors declare that the research was conducted in the absence of any commercial or financial relationships that could be construed as a potential conflict of interest.

## Footnotes

1 https://www.ncbi.nlm.nih.gov/Traces/study/?acc=PRJNA607375

2 http://www.bioinformatics.babraham.ac.uk/projects/fastqc/

3 http://circrna.org/

## Abbreviations

α -SMA: smooth muscle actin- α
AS: Atherosclerosis
ceRNAs: competitive endogenous RNAs
circRNA: circular RNA
DE circRNAs: differentially expressed circRNAs
ECM: extracellular matrix
miRNA: microRNA
NC group: the blank control group did not receive PDGF-BB
ncRNAs: noncoding non-coding RNAs
PS group: Cells were treated with PDGF-BB
SM22 α: smooth muscle 22 α
VSMCs: Vascular smooth muscle cells.
