# Supplementary figures and images for: Differential Expression and Bioinformatics Analysis of CircRNA in PDGF-BB-Induced Vascular Smooth Muscle Cells
Source: Front Genet. 2020 May 29;11:530. doi: 10.3389/fgene.2020.00530 (PMC7272660; doi:10.3389/fgene.2020.00530)

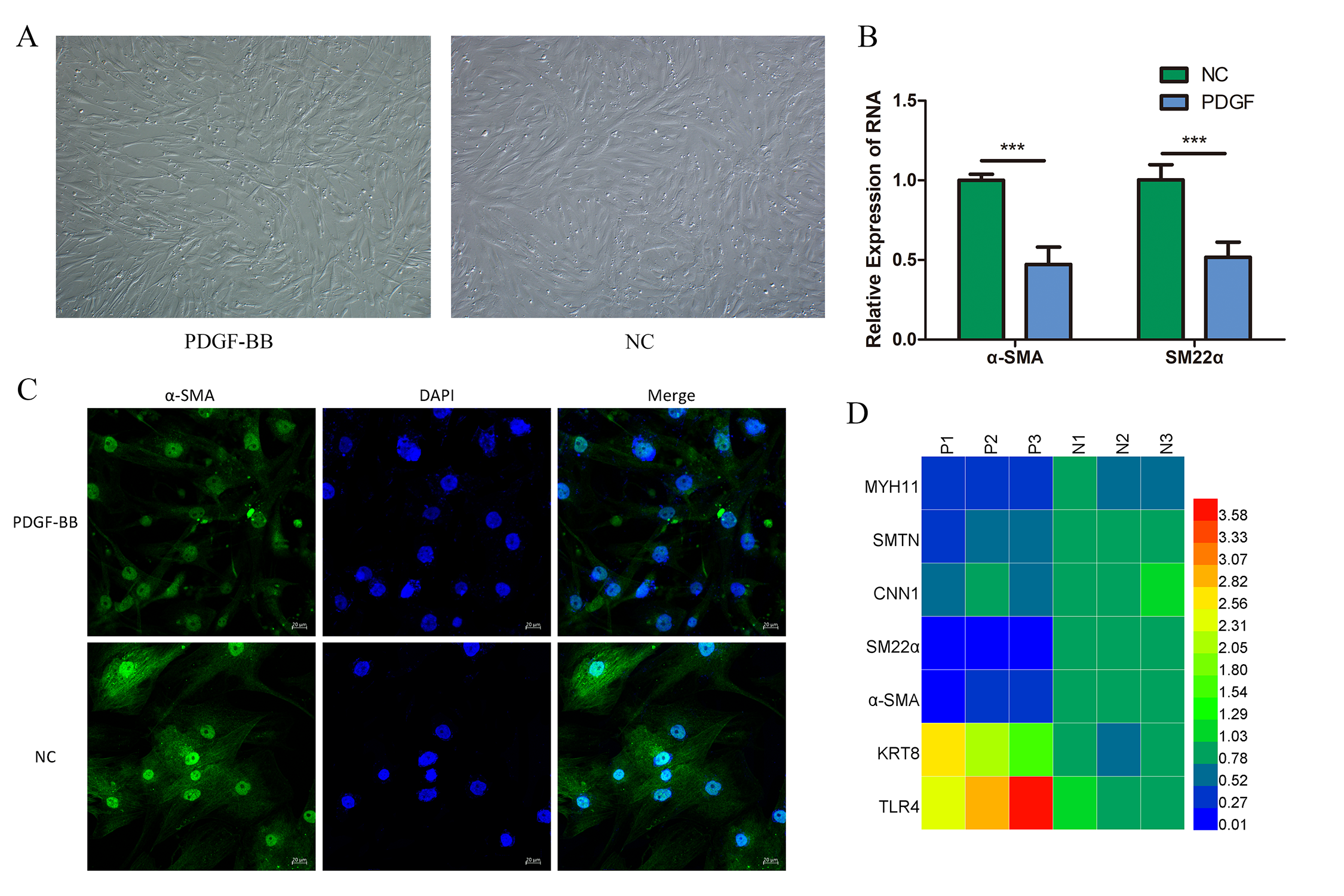

Supplement: Supplementary file 1 [file Image_1.TIF]

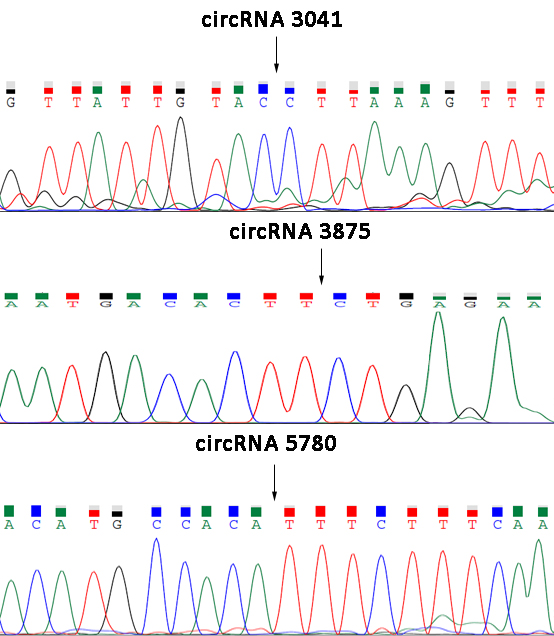

Supplement: Supplementary file 2 [file Image_2.TIF]
