# Supplementary material for: Differential Expression and Bioinformatics Analysis of CircRNA in PDGF-BB-Induced Vascular Smooth Muscle Cells
Source: Front Genet. 2020 May 29;11:530. doi: 10.3389/fgene.2020.00530 (PMC7272660; doi:10.3389/fgene.2020.00530)
Supplement: Supplementary file 3 [file Table_1.DOCX]

**supplementary Table1: Primers used in RT-PCR**

| **Name** | **Forward primer (5' to 3')** | **Reverse primer (5' to 3')** |
| --- | --- | --- |
| GAPDH | AAGGTCGGAGTCAACGGATT | ATGGAATTTGCCATGGGTGG |
| MYH11 | GCATGCTTCAAGATCGGGAG | TCTCCCGTGATACTTGTGTCT |
| SMTN | AGCTGGAGTCCATGAACGAT | CCCTTGCTGTCCTCTCTTGA |
| CNN1 | TTTTTGAGGCCAACGACCTG | TGATGTTCCGCCCTTCTCTT |
| SM22α | TGGTGAACAGCCTGTACCCT | CACGGTAGTGCCCATCATTC |
| α-SMA | GGGTGATGGTGGGAATGG | GCAGGGTGGGATGCTCTT |
| KRT8 | ATCAGCTCCTCGAGCTTCTC | CCAGGAACCGTACCTTGTCT |
| TLR-4 | GCGTGGAGGTGGTTCCTAA | ACTCTGGATGGGGTTTCCTG |
| circRNA4452 | GTGGCCAGTACAAAGACACG | AGTATCTGCTGTCTCACCTGA |
| circRNA13360 | CGAAGTTCTTCAAGAGGGCG | CTCGGAGCCTACATCTTTTCG |
| circRNA1698 | GCCTCTCAGAACCAGAACCT | AGTGAATCTTCTTAGTGGTTCCA |
| circRNA8979 | GGAGATCGTGCAGGTGTAGT | AGGGGAAGAAGTTGGCGTAG |
| circRNA14411 | TCCTCTCACAGCCATTCCAG | CTGGCAATGTTTCCATCTCGT |
| circRNA3041 | GATAAAACTTTAAGGTACAATAACTTGCC | GCATTCCCATTTCTTTGCATCC |
| circRNA1848 | TGTTTTAAGCTTCATCAGCAATGG | CTGGCATCTCAACATTCGGG |
| circRNA5780 | TGGCAGTGAAAAGAAGGGGT | TGAAAGAAATGTGGCATGTGAGA |
| circRNA536 | GCACGAACACCAAGGAACAT | TCTCAGGCATCACAGTGGTT |
| circRNA2637(◄►) | GTTCTTCTTTGGATGACACCGA | TGCCTCCTTCCACTTGAACT |
| circRNA2637(►◄) | CCACAACTGCATCAAAGACAGAA | TCACCTTGCAGCCTCTTACAT |
| circRNA4624(◄►) | AGGATAATGCCCTGCTGACA | TGGATGGGTTTAACCTCCACTT |
| circRNA4624(►◄) | AAATCCCCAAATGTGCTGCC | ACCAACACTCTGCAGTGCTA |
| circRNA4487(◄►) | CGGAGTGCTGTTGGAAGTTC | CGAAGAAAGTTATACAGCTGTGG |
| circRNA4487(►◄) | GAGCAAGTGCAGCAAAGTAGA | AGTATTGAACTTCCAACAGCACT |
| circRNA3875(◄►) | AACACGCTTTGGAAAAGGCA | GCTGCAACAATGACACTTCTG |
| circRNA3875(►◄) | AAGTCAGCAGTCTCACCTTCCA | TGGGCTGAATCTACTGAGGAAA |
| circRNA4209(◄►) | CAGTGCATTCAAGGAAGCCA | TTCAGCAATGGTGGCAGTTC |
| circRNA4209(►◄) | ACAATGATGATGACCGACCAAA | TTCACCAAGTGGGGCATCA |
| circRNA5591(◄►) | CAATGGTGGATGCCCTGATG | AGGTCCTCAGGGATGTTATCTT |
| circRNA5591(►◄) | CTGTCCCGGAAAGGGATCTA | TTTTATGGGTGCGCGACTG |
| circRNA5550(◄►) | GCCAGATTTGCATAAGGCTGA | CCCTGAATTCCTGGTGGTCT |
| circRNA5550(►◄) | CATTTGCCCAGAAACGTCGG | CTTATGCAAATCTGGCATCAAATCC |
| circRNA5497(◄►) | TGCTTGGTGGACGTCTGATT | AGCTGTGTACCTGATGCTGT |
| circRNA5497(►◄) | GCACCCCTCTACAGTGACTC | TCATGGTATCCCGATTCCGC |
| circRNA5223(◄►) | CATCACTACCGGACCCAGAG | GTCGTAGCAGGTCATCTCCA |
| circRNA5223(►◄) | AATCCCTGGACTCGGATGAG | TCATCATCCTCCGTCATGGT |
| RPL13A(◄►) | AAGCCAAGATCCACTACCGG | TGTCACTGCCTGGTACTTCC |
| RPL13A(►◄) | AGAATGTGCAAGCACTTGGG | CTGTAACCCCTTGGTTGTGC |
